# Neuronal and Glial Cultures Differentiated from iPSCs of Healthy Donors and Patients with *PARK2*-Associated Parkinson’s Disease Exhibit Distinct Expression Patterns of Receptors for Resolvin E1 and Resolvin D1

**DOI:** 10.3390/brainsci16101047

**Published:** 2026-09-29

**Authors:** Tatiana Gerasimova, Ekaterina Novosadova, Ekaterina Stepanenko, Lyudmila Novosadova, Elena Arsenyeva, Artem Eremeev, Valentina Nenasheva

**Affiliations:** 1 Laboratory of Translational Biomedicine, Lopukhin Federal Research and Clinical Center of Physical–Chemical Medicine of Federal Medical Biological Agency, 1A Malaya Pirogovskaya Str., 119435 Moscow, Russia; novek-img@mail.ru (E.N.); katishsha@mail.ru (E.S.); val-nenasheva@mail.ru (V.N.); 2 Laboratory of Molecular Neurogenetics and Innate Immunity, National Research Centre “Kurchatov Institute”, 2, Akad. Kurchatova Sq., 123182 Moscow, Russia; novosadova-l@rambler.ru (L.N.); arslena@mail.ru (E.A.); 3 Center for Genetic Reprogramming and Gene Therapy, Lopukhin Federal Research and Clinical Center of Physical-Chemical Medicine of Federal Medical Biological Agency, 1A Malaya Pirogovskaya Str., 119435 Moscow, Russia

**Keywords:** Parkinson’s disease, *PARK2* mutations, neuroinflammation, neuron, glia, resolvin E1, resolvin D1, receptors

## Abstract

**Highlights:**

**What are the main findings?**

- Neurons and glia with different *PARK2* mutations express receptors for RvE1 and RvD1 at both the mRNA and protein levels and have inflammatory gene activity distinct from HD cells.
- Compared with HD neurons, *PARK2*-mutant neurons showed similar *CHEMR23* mRNA levels but significantly higher CHEMR23 protein expression.
- In a pilot experiment, RvD1 affected IL-6 protein secretion and cellular metabolic activity in the examined glial cell lines.

**What are the implications of the main findings?**

- Future studies aimed at developing resolvin-based therapeutic strategies for genetic forms of Parkinson’s disease should take into account the mutation-specific inflammatory status and resolvin receptor expression profiles in different cell types.
- Resolvin D1 warrants further investigation for its potential therapeutic effects in PD associated with *PARK2* mutations.

**Abstract:**

**Background:** Neuroinflammation and neurodegeneration are closely linked in Parkinson’s disease (PD). Specialized pro-resolving mediators (SPMs) are of interest as potential therapeutic agents for PD because they promote the resolution of inflammation. **Methods:** We investigated whether *PARK2* mutations are associated with cell-type-specific patterns of SPM receptor expression by comparing neuronal and glial cultures derived from induced pluripotent stem cells (iPSCs) of PD patients carrying different *PARK2* mutations with corresponding cultures from healthy donors (HDs), using real-time PCR and Western blotting. **Results:** The resolvin E1 receptors, BLT1 and CHEMR23, and the resolvin D1 (RvD1) receptors, GPR32 and FPR2, were detected at both the mRNA and protein levels in *PARK2*-mutant neurons and glia. Compared with HD cultures, mutant neurons had significantly higher CHEMR23 protein levels despite similar *CHEMR23* mRNA levels, whereas no statistically detectable differences in the expression of any of the four receptors were found in glia. Receptor profiles also appeared to differ between cells carrying *PARK2* mutations and those carrying mutations in other genes (*GBA1*, *LRRK2*). *PARK2*-mutant neurons showed lower levels of several inflammatory markers than HD neurons, in contrast to the pro-inflammatory profile previously reported in *PARK2*-mutant glia. A pilot experiment with glial cultures derived from one cell line per group suggested that in the presence of TNFα, RvD1 may reduce IL-6 secretion and increase cellular metabolic activity in *PARK2*-mutant glia, with responses that appeared to differ from those observed in HD glia. **Discussion:** The presence of resolvin receptors in *PARK2*-mutant neurons and glia, together with elevated CHEMR23 protein levels in mutant neurons compared with HD neurons, provides a basis for further investigation of resolvin signaling in *PARK2*-associated PD. However, the relationship between receptor expression and functional responses to resolvins remains to be established. The contrasting inflammatory profiles of neurons and glia suggest that *PARK2*-mutant glial cultures may be particularly relevant for investigating the anti-inflammatory effects of resolvins. **Conclusions:** Cell-type- and genetic-background-associated differences in receptor expression should be considered when developing resolvin-based therapeutic approaches for PD. Further studies are needed to assess the potential anti-inflammatory effects of RvD1 on glial cells in genetic forms of the disease.

## 1. Introduction

From a biological perspective, inflammation is an adaptive response to damaging agents aimed at restoring tissue homeostasis. Acute inflammation is self-limited and serves as a protective host reaction. In contrast, long-lasting chronic inflammation can lead to detrimental consequences and is involved in the pathogenesis of several complex diseases, such as atherosclerosis, diabetes mellitus, chronic obstructive pulmonary disease, multiple sclerosis, Crohn’s disease, ulcerative colitis, chronic periodontitis, asthma, renal fibrosis, and neurodegeneration, including Parkinson’s disease (PD) [1,2,3,4,5,6].

It has been proposed that specialized mechanisms exist to minimize inflammatory tissue damage while preserving the protective aspects of inflammation [7]. Such mechanisms were discovered by Serhan et al., which changed the paradigm of inflammation by highlighting an active, well-coordinated program of resolution [2,8,9]. The resolution phase is characterized by a shift in the arachidonic acid cascade from synthesis of inflammatory prostaglandins and leukotrienes to anti-inflammatory lipoxins, as well as the conversion of omega-3 polyunsaturated fatty acids (PUFAs) into resolvins (Rv) and protectins. These molecules, members of the superfamily of specialized pro-resolving lipid mediators (SPMs), are key mediators in the resolution of inflammation and exhibit anti-inflammatory activity without inducing immunosuppression.

Patients with PD have an increased inflammatory status. Concentrations of pro-inflammatory cytokines and chemokines have been found to be upregulated in serum, cerebrospinal fluid, and postmortem brain tissue samples from PD patients [10,11]. The death of dopaminergic neurons in the substantia nigra, along with the accumulation of modified α-synuclein, perpetuates chronic inflammation in brain tissue. The main source of inflammatory mediators is the microglial and astrocytic inflammatory response [12]. On one hand, excessive amounts of inflammatory mediators disrupt neuronal function, contributing to the progression of neurodegeneration. On the other hand, insufficient effectiveness of the resolution program and altered SPM function are also characteristic to neurodegeneration, since SPM levels in striatum and SN of PD patients are downregulated [12,13]. Thus, an imbalance between neuroinflammatory and pro-resolving processes is established in PD [14].

The functions of SPMs in the central nervous system have been investigated in cellular and animal experimental models of PD [15,16] primarily focusing on microglia and astrocytes [13]. Data on the effects of these molecules on neurons, traditionally considered inert victims of immune response, are scarce. However, evidence is gradually accumulating regarding the ability of neurons to express key immune molecules, including Toll-like and cytokine receptors, complement molecules, and components of main inflammatory signaling pathways [17,18,19]. This indicates that neurons have the capacity to respond to the inflammatory environment depending on the profile of surrounding mediators, including resolvins. Additionally, astrocytes can release PUFAs, including docosahexaenoic acid (DHA), which serves as a precursor for SPM biosynthesis [20]. Immunohistochemical studies of human brain tissue have shown that neurons and glial cells express 5-LOX and 12/15-LOX, enzymes involved in lipid mediator biosynthesis [21,22]. Together with the evidence for SPM receptor expression, these findings suggest that both cell types may participate in SPM biosynthesis and signaling.

Epidemiological studies have also reported an association between the use of some anti-inflammatory drugs and a reduced risk of developing PD [23]. PD-associated genes, including the *PARK2* gene encoding the E3 ubiquitin-protein ligase Parkin, may also play an important role in neuroinflammation [18]. Recent studies have demonstrated a link between *PARK2* mutations with “mito-inflammation” resulting from mitochondrial dysfunction [4]. Another report points to the direct involvement of Parkin in intracellular inflammatory signaling pathways [24].

We conducted the present study to determine the expression of specific receptors for SPMs in iPSC-derived neurons and glial cells from PD patients with different mutations in the *PARK2* gene, in comparison to healthy donors (HDs). Given the involvement of Parkin in the immune response and the clear association of the *PARK2* gene with PD, we propose that the expression of receptors for certain SPMs in cells with *PARK2* mutations may exhibit specific characteristics.

## 2. Materials and Methods

### 2.1. iPSC Differentiation in Neuronal and Glial Direction

The characteristics of all of the cell lines used in this study are given in Table 1.

The differentiation of iPSCs (in glial and neuronal direction) was performed as described earlier [25,26]. Once the iPSC cultures reached 60–80% confluence, the iPSC maintenance medium, StemMACS XF (Miltenyi Biotec, Auburn, CA, USA), was replaced with Neurobasal Medium (Thermo Fisher Scientific, Grand Island, NY, USA) supplemented with a cocktail of factors promoting neural differentiation: Noggin, 80 ng/mL (Gibco, Grand Island, NY, USA); 1% B-27 and 1% N-2 (both Gibco, Grand Island, NY, USA); and SB431542, 10 μM (Selleck Chemicals, Houston, TX, USA). After 10–14 days of culture, the resulting neural rosettes and ridges were mechanically transferred to 24-well ultra-low-attachment plates (Corning Costar, Corning, NY, USA) to induce neurosphere formation. After 5–7 days, the neurospheres were trypsinized to obtain a single-cell suspension and transferred to Petri dishes pre-coated with Matrigel (Corning, Corning, NY, USA). The cells were then cultured in neural progenitor (NP) medium for 14 days. Differentiation of the resulting NPs toward the neuronal lineage was performed using a two-stage protocol. (i) Regional specification. Uncommitted NPs at passages 2–4 were transferred to Neurobasal Medium supplemented with Shh, 100 ng/mL (PeproTech, Rocky Hill, NJ, USA); FGF8, 20 ng/mL (PeproTech, Rocky Hill, NJ, USA); and Purmorphamine, 10 μM (Stemgent, Cambridge, MA, USA) for 10 days. (ii) Generation of differentiated neurons: Shh, FGF8, and Pur were replaced in the culture medium with GDNF, 20 ng/mL and BDNF, 20 ng/mL (both PeproTech, Cranbury, NJ, USA); Forskolin, 4 μM (Stemgent, Cambridge, MA, USA); and ascorbic acid, 200 μM (PanEco, Moscow, RF). For differentiation toward the glial lineage, the same NPs at passages 2–4 were transferred to Neurobasal Medium supplemented with FGF2, 10 ng/mL (Gibco, Grand Island, NY, USA); heregulin, 200 ng/mL (Thermo Fisher Scientific, Waltham, MA, USA); IGF-1, 10 ng/mL (ProSpec, East Brunswick, NJ, USA); activin A, 10 ng/mL (Gibco, Grand Island, NY, USA), N-2 (1%) and B-27 (1%) for 28 days. For the pilot experiment with resolvin D1, IPSHD1.1S and IPSPDS14 glial cells were seeded at a density of 7000 cells per well in a 96-well plate and cultured in the presence or absence of RvD1, 20 ng/mL (MedChemExpress, Monmouth Junction, NJ, USA) for 24 h, both under unstimulated conditions and following the induction of inflammation with TNF-α, 10 ng/mL (#PSG250-10, SCI-store, Moscow, RF).

### 2.2. Immunocytochemistry

**Figure 1:**
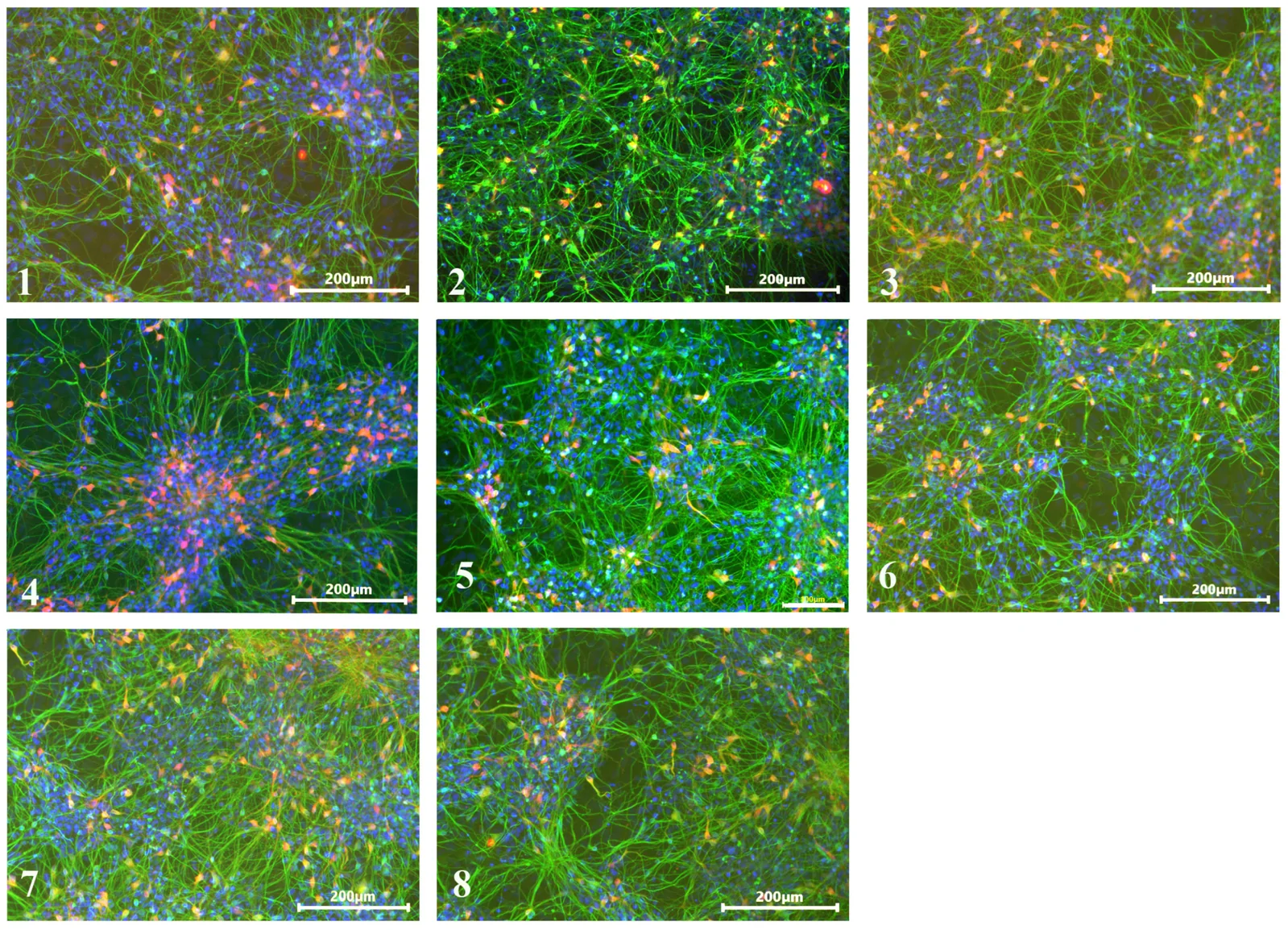
Immunocytochemical staining of neurons with antibodies against the pan-neuronal marker TUBB3 (green), the dopaminergic neuron marker tyrosine hydroxylase (red), and the nuclear dye DAPI (blue). Cell lines: **1**—IPSRG2L, **2**—IPSHD1.1S, **3**—IPSFD3.9L, **4**—IPSPDS12, **5**—IPSPDS13, **6**—IPSPDS14, **7**—IPSPDSG1.1, **8**—IPSPDL2.15L. Scale bar: 100 µm for panel 5 and 200 µm for all other panels.

**Figure 2:**
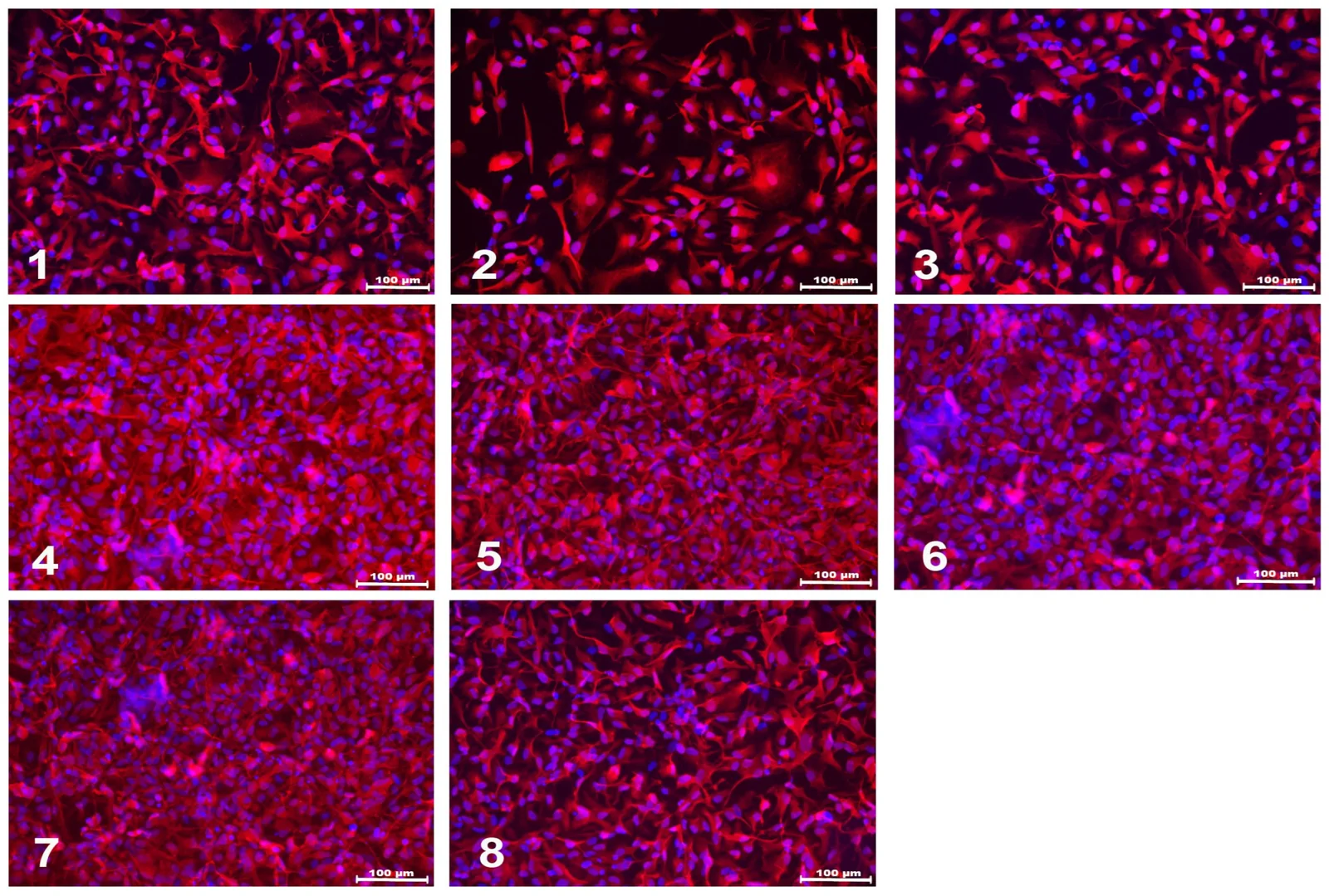
Immunocytochemical staining of glial cells with antibodies against S100 (red) and the nuclear dye DAPI (blue). Cell lines: **1**—IPSRG2L, **2**—IPSHD1.1S, **3**—IPSFD3.9L, **4**—IPSPDS12, **5**—IPSPDS13, **6**—IPSPDS14, **7**—IPSPDSG1.1, **8**—IPSPDL2.15L. Scale bar: 100 µm.

Following completion of differentiation, iPSC-derived neuronal and glial cultures were characterized by immunofluorescence staining for the cell-type-specific markers TUBB3/TH and S100, respectively (Figure 1 and Figure 2). Cells grown adherently in Petri dishes were rinsed with PBS and fixed in 4% paraformaldehyde prepared in PBS (pH 6.8) for 30 min at room temperature (RT). Following fixation, the cultures were washed three times for 5 min with PBS containing 0.1% Tween 20 (Sigma-Aldrich, Saint Louis, MO, USA). To reduce nonspecific antibody binding, the cells were incubated for 30 min at RT in blocking buffer consisting of PBS supplemented with 0.1% Triton X-100 and 5% fetal bovine serum (HyClone, Logan, UT, USA). The cultures were then incubated overnight at 4 °C with either mouse anti-TUBB3 (1:2000, AB7751, Abcam, Waltham, MA, USA) and rabbit anti-TH (1:1000, AB112, Abcam, Waltham, MA, USA), or rabbit anti-S100 (1:2, GA50461-2, Agilent Dako, Santa Clara, CA, USA) primary antibodies. After three 5 min washes with PBS containing 0.1% Tween 20, the corresponding secondary antibodies, goat anti-mouse (1:1000, A11008, Thermo Fisher, Waltham, MA, USA) or goat anti-rabbit (1:1000, A11010, Thermo Fisher, Waltham, MA, USA), were applied for 60 min at RT. The cultures were subsequently washed three times for 5 min with PBS containing 0.1% Tween 20. Cell nuclei were counterstained with 0.1 μg/mL DAPI (Sigma-Aldrich, Saint Louis, MO, USA) in PBS for 10 min, followed by two washes with PBS. Fluorescence imaging was performed using an AxioImager Z1 fluorescence microscope (Carl Zeiss, Oberkochen, Germany), and images were acquired using AxioVision release 4.8.1 (11-2009) software (Carl Zeiss, Oberkochen, Germany).

### 2.3. Quantitative Real-Time PCR (qPCR)

Total RNA was isolated from cell cultures using TriReagent (MRC, Cincinnati, OH, USA) according to the manufacturer’s protocol. Residual genomic DNA was subsequently removed using the DNA-free DNA Removal Kit (Invitrogen, Carlsbad, CA, USA). For cDNA synthesis, 0.5–2 μg of total RNA was reverse-transcribed with M-MLV Reverse Transcriptase (Evrogen, Moscow, RF, Russia) using random primers. Primer sequences are provided in Table 2 and have been reported previously [27]. Quantitative PCR was performed using qPCRmix-HS SYBR (Evrogen, Moscow, RF, Russia) on a LightCycler 96 instrument (Roche, Basel, Switzerland). The amplification protocol consisted of an initial denaturation (preincubation) at 95 °C for 5 min, followed by 45 amplification cycles at 94 °C for 20 s, 55–65 °C for 20 s, and 72 °C for 20 s. Melting curve analysis was performed at 95 °C for 10 s, 65 °C for 60 s, and 97 °C for 1 s, followed by cooling at 37 °C for 30 s. *18S* rRNA was used as the reference gene for glial cultures, whereas *HMBS* mRNA was used for neuronal cultures based on previously published recommendations [28]. The use of a single reference gene for normalization should be taken into consideration when interpreting the qPCR results. Relative changes in target gene expression were determined using the 2^−ΔΔCt^ method and expressed as fold change (FC).

### 2.4. Western Blot Hybridization

Cell and tissue samples were lysed in RIPA buffer (Thermo Fisher Scientific, Waltham, MA, USA) supplemented with a protease inhibitor mixture (Sigma-Aldrich, St. Louis, MO, USA). Total protein concentration was measured using the BCA assay. For each sample, 20 μg of total protein was subjected to SDS-polyacrylamide gel electrophoresis (SDS-PAGE), followed by transfer onto a PVDF membrane (Bio-Rad, Hercules, CA, USA). Membranes were blocked with 5% milk and subsequently incubated overnight at 4 °C with primary antibodies diluted in 1% milk: rabbit anti-BLT1 (1:500, #DF10223), rabbit anti-CHEMR23 (1:1000, #DF2712), rabbit anti-GPR32 (1:500, #DF2719), rabbit anti-FPRL1 (1:750, #DF4971) (all from Affinity Biosciences, Cincinnati, OH, USA), or mouse anti-β-actin (1:6000, #A5441, Sigma-Aldrich, Burlington, MA, USA). After primary antibody incubation, the membranes were washed five times for 5 min with Tris–NaCl–Tween (TNT) buffer and incubated for 2 h at RT with the appropriate horseradish peroxidase (HRP)-conjugated secondary antibody diluted in 1% milk (1:10,000, #31466, Thermo Fisher Scientific, Waltham, MA, USA; or 1:8000, #140777, Jackson ImmunoResearch, Cambridgeshire, UK). After washing five times for 5 min with TNT, protein signals were detected using an enhanced chemiluminescence reagent (#170-5061, Bio-Rad, Hercules, CA, USA) and visualized with the ChemiDoc MP Imaging System (Bio-Rad, Hercules, CA, USA).

### 2.5. ELISA

IL-6 protein levels in cell culture supernatants were quantified using a human IL-6 ELISA kit (cat. #A-8762, Vector-Best, Moscow, RF) in accordance with the manufacturer’s instructions. Absorbance was measured at 450 nm using a Metertech microplate reader (Nankang, Taipei, Taiwan). The measured IL-6 concentrations were normalized to the number of cells in each well and expressed as the amount per 1 × 10^3^ cells.

### 2.6. Statistical Analysis

Statistical analysis of the data was performed using GraphPad Prism version 10.4.0. (build 621; GraphPad Software, Boston, MA, USA) software. The data from 1 to 3 independent differentiation experiments for each cell line were obtained, and the mean value for each cell line was included in pairwise comparison, to avoid disproportionate contribution of any single cell line. All data was normalized to the mean value of the healthy donors (HD) group; differences between groups were expressed as fold changes. Results were presented as mean ± SD. Data distribution was assessed for normality using the Shapiro–Wilk test. For normally distributed data, an unpaired *t*-test was used for pairwise comparisons. The differences were considered statistically significant at * *p* < 0.05, ** *p* < 0.01. The pilot experiment on the effects of RvD1 on glial cells was analyzed separately, and since only one HD and one *PARK2*-mutant cell line were used, no statistical comparisons between the HD and *PARK2*-mutant cell lines were performed.

## 3. Results

According to the literature data, several SPM receptors are expressed in neurons: CHEMR23 [29], GPR32 [30], FPR2 [31], and in astrocytes: CHEMR23, BLT1, and GPR37 [13]. The protein product of the latter is a Parkin substrate for ubiquitination. The evidence exists that in the case of Parkin loss, GPR37 forms insoluble aggregates and triggers the unfolded protein response [32]. Similarly to α-synuclein, the protein has been found in the Lewy bodies of PD patients [33,34,35]. We hypothesized that in cell cultures with *PARK2* mutations GPR37 might function incorrectly regardless of the expression level and, therefore, excluded the receptor from further analysis and focused on RvE1 and RvD1 receptors.

**Figure 3:**
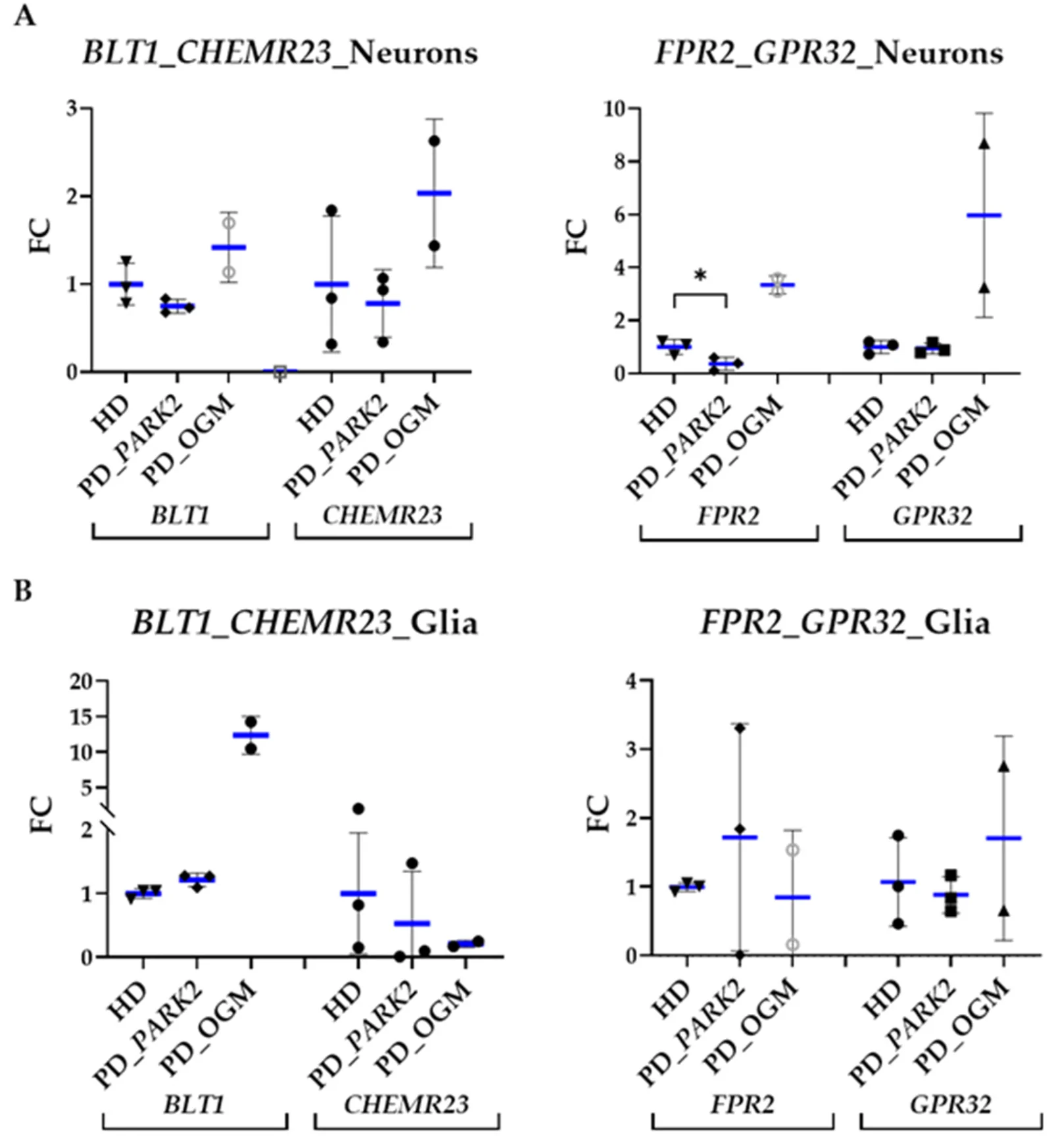
Expression of genes encoding receptors for RvE1 (BLT1, CHEMR23) and RvD1 (GPR32, FPR2) in the cell cultures of patients with *PARK2*-associated PD (PD_*PARK2*) compared with HD cells and the group carrying PD-associated mutations in other genes (PD_OGM). Cell lines: 1—IPSRG2L, 2—IPSHD1.1S, 3—IPSFD3.9L, 4—IPSPDS12, 5—IPSPDS13, 6—IPSPDS14, 7—IPSPDSG1.1, 8—IPSPDL2.15L. qPCR. (**A**) neurons, ref. *HMBS* mRNA, FC (fold change) = gene expression/mean of gene expression in HD cells; (**B**) glial cells, ref *18S* rRNA, FC. Each data point represents the mean value obtained for an individual cell line across independent differentiations. The results are presented as mean ± SD, with the mean value for the group indicated in blue. For normally distributed data, statistical analysis was performed using an unpaired *t*-test (GraphPad Prism 10.4.0. software). The differences were considered statistically significant at * *p* < 0.05.

qPCR analysis showed that all four resolvin receptor genes were expressed in both neuronal and glial cells. Overall, the expression levels of all four receptors in *PARK2*-mutant neurons (PD_*PARK2*) did not exceed those observed in HD neurons. The only statistically significant difference was a decrease in *FPR2* expression. To explore whether this expression pattern might be specifically associated with the *PARK2* genetic background, we additionally compared *PARK2*-mutant cells with a group carrying PD-associated mutations in other genes (PD_OGM), which included neuronal and glial cell lines with mutations in LRRK2 (*PARK8*) and *GBA1*. In contrast to PD_*PARK2* neurons, PD_OGM neurons showed a general tendency toward higher expression of all four receptor genes compared with HD cells. In *PARK2*-mutant glial cells, no significant changes in resolvin receptor gene expression were observed compared with HD cells, whereas PD_OGM glia showed a tendency toward higher *BLT1* and lower *CHEMR23* expression. Although these changes did not reach statistical significance, the observed trends suggest that resolvin receptor expression may vary depending on the underlying PD-associated mutation (Figure 3).

Next, we investigated the pattern of the expression of RvE1 receptors (BLT1, CHEMR23) and RvD1 receptors (GPR32, FPR2) in *PARK2*-mutant and HD neurons and glial cells at the protein level.

**Figure 4:**
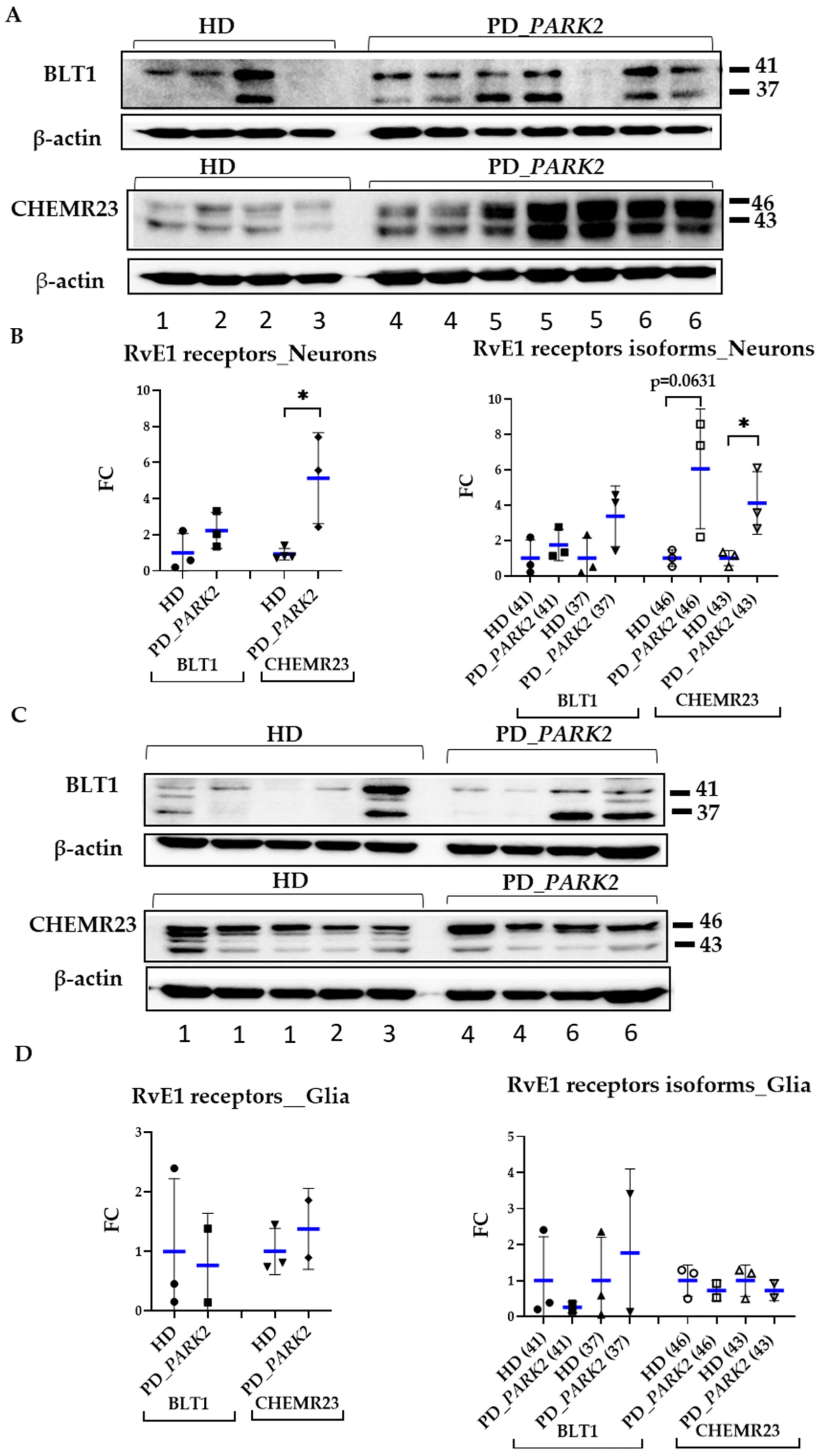
Expression of receptors for RvE1 (BLT1, CHEMR23) at the protein level in the cell cultures of patients with *PARK2*-associated PD compared with those of healthy donors. Cell lines: 1—IPSRG2L, 2—IPSHD1.1S, 3—IPSFD3.9L, 4—IPSPDS12, 5—IPSPDS13, 6—IPSPDS14. Western blot hybridization in neurons (**A**) and glia (**C**), reference protein β-actin. Relative expression of the detected receptor bands in neurons (**B**) and glial cells (**D**). FC (fold change) = protein expression in cell culture/mean of protein expression in HD cells. Each data point represents the mean value obtained for an individual cell line across independent differentiations. The results are presented as mean ± SD, with the mean value for the group indicated in blue. For normally distributed data, statistical analysis was performed using an unpaired *t*-test (GraphPad Prism 10.4.0. software). The differences were considered statistically significant at * *p* < 0.05.

RvE1 receptors were detected in both cell types as two closely migrating protein bands: one at approximately 37 kDa for BLT1 and 43 kDa for CHEMR23, and another approximately 3–4 kDa higher, which may represent a modified form of the protein. Evidence exists that phosphorylation of BLT1 promotes receptor desensitization [36]. The main finding was that in *PARK2*-mutant neurons, CHEMR23 protein was present at a significantly higher level, compared to HD cells (Figure 4).

**Figure 5:**
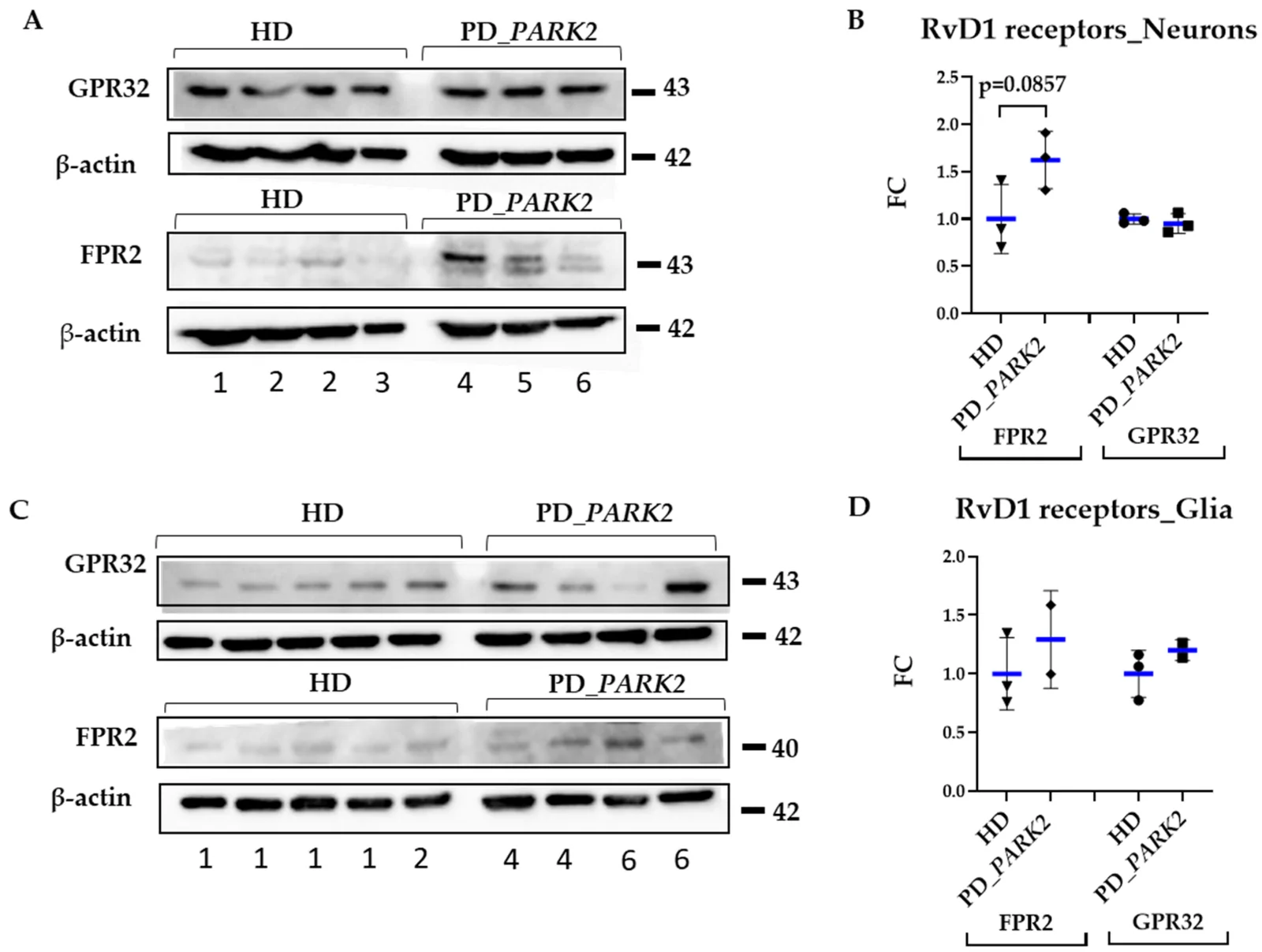
Expression of receptors for RvD1 (GPR32, FPR2) at the protein level in the cell cultures of patients with *PARK2*-associated PD compared to those of healthy donors. Cell lines: 1—IPSRG2L, 2—IPSHD1.1S, 3—IPSFD3.9L, 4—IPSPDS12, 5—IPSPDS13, 6—IPSPDS14. Western blot hybridization in neurons (**A**,**B**) and glia (**C**,**D**), reference protein β-actin. FC (fold change) = protein expression in cell culture/mean of protein expression in HD cells. Each data point represents the mean value obtained for an individual cell line across independent differentiations. The results are presented as mean ± SD, with the mean value for the group indicated in blue. For normally distributed data, statistical analysis was performed using an unpaired *t*-test (GraphPad Prism 10.4.0. software).

Expression of receptors for RvD1 (GPR32 and FPR2 proteins) was also detected both in neurons and glial cultures. Compared with the corresponding HD cells, FPR2 protein levels showed a tendency to be higher in *PARK2*-mutant neurons (Figure 5).

**Figure 6:**
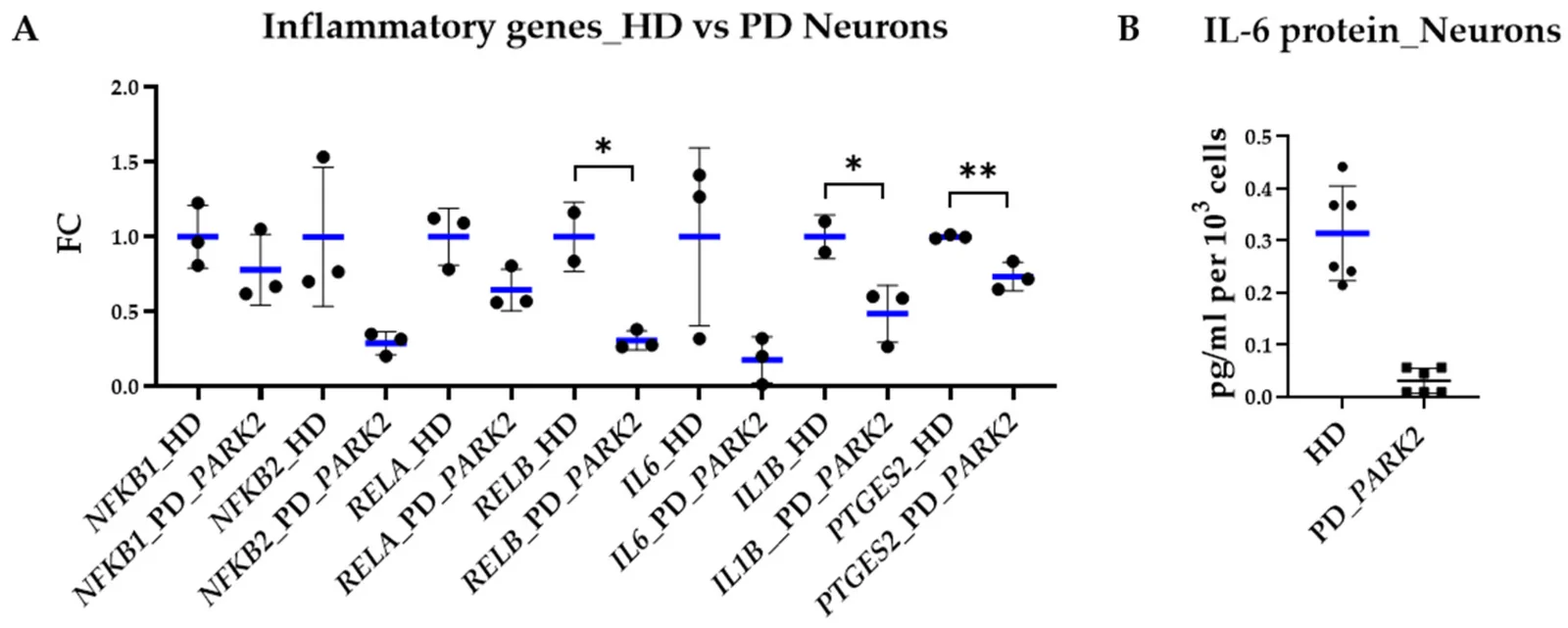
Expression of the inflammatory genes in neuronal cultures of patients with *PARK2*-associated PD compared to those of HD. Cell lines: 1—IPSRG2L, 2—IPSHD1.1S, 3—IPSFD3.9L, 4—IPSPDS12, 5—IPSPDS13, 6—IPSPDS14. (**A**) expression of the inflammatory genes, qPCR, ref. *HMBS* mRNA, FC (fold change) = gene expression/mean of gene expression in HD. The results are presented as mean ± SD. For normally distributed data, statistical analysis was performed using an unpaired *t*-test (GraphPad Prism 10.4.0. software). Differences were considered statistically significant at * *p* < 0.05, ** *p* < 0.01. (**B**) IL-6 protein level in two HD (IPSRG2L, IPSHD1.1S) vs. two *PARK2*-mutant (IPSPDS12, IPSPDS14) neuronal cell culture supernatants, ELISA. Data are from three independent experiments performed with each cell line and are presented as mean ± SD, with the mean value for the group indicated in blue. Statistical analysis was not performed because of the limited number of cell lines in both groups.

To assess how these findings corresponded to the overall inflammatory status of both cell types, we determined the expression of genes encoding components of NF-κB and the main pro-inflammatory cytokines IL1B and IL6, since their activity is a result of the network of various inflammatory signaling pathways. We also estimated the level of PTGES2 encoding microsomal prostaglandin E synthase-2 (mPGES-2), one of the enzymes that convert PGH_2_ into strong pro-inflammatory PGE_2_. We found that several of the studied genes (*RELB*, *IL1B*, *PTGES2*) were downregulated in PD_*PARK2* neurons compared with HD neurons (Figure 6A). ELISA measurements of IL-6 protein levels revealed trends consistent with the qPCR data (Figure 6B). In a recent study we reported the opposite pattern characteristic for *PARK2*-mutant glial cultures as they exhibited a more pro-inflammatory status compared to HD [27].

**Figure 7:**
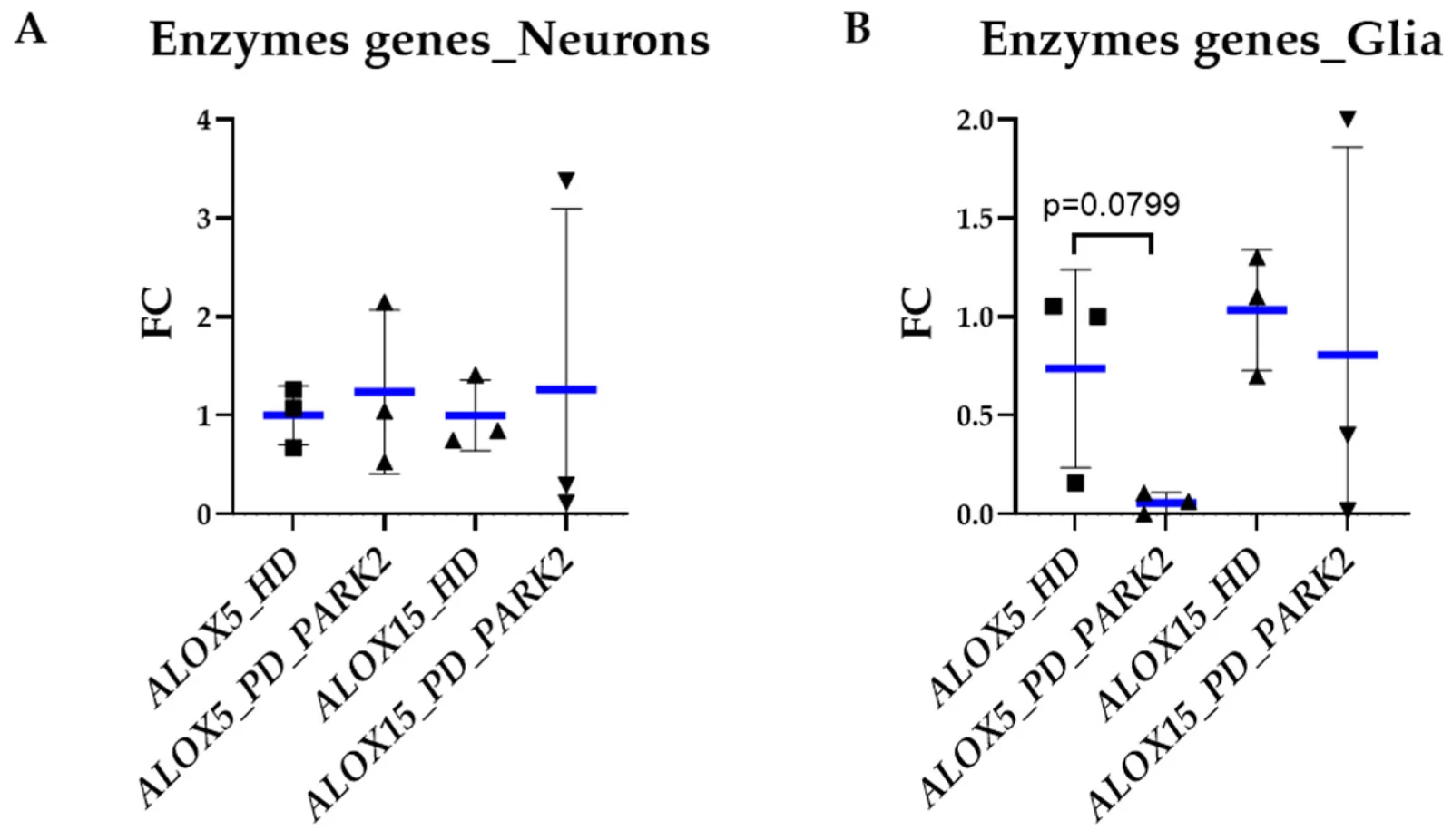
Expression of genes encoding enzymes involved in resolvin synthesis in cell cultures from patients with *PARK2*-associated PD compared with healthy donors. Cell lines: 1—IPSRG2L, 2—IPSHD1.1S, 3—IPSFD3.9L, 4—IPSPDS12, 5—IPSPDS13, 6—IPSPDS14. (**A**) neurons, qPCR, ref. *HMBS* mRNA, FC (fold change) = gene expression/mean of gene expression in HD; (**B**) glial cell cultures, qPCR, ref *18S* rRNA, FC. Each data point represents the mean value obtained for an individual cell line across independent differentiations. The results are presented as mean ± SD, with the mean value for the group indicated in blue. For normally distributed data, statistical analysis was performed using an unpaired *t*-test (GraphPad Prism 10.4.0. software).

In order to assess the expression of genes involved in endogenous SPM biosynthesis in *PARK2*-mutant neurons and glial cells, we analyzed *ALOX5* (encoding 5-lipoxygenase) and *ALOX15* (encoding 15-lipoxygenase) [37]. Both genes were expressed in neuronal and glial cultures, with no significant differences between *PARK2*-mutant and HD cells, although *ALOX5* showed a tendency toward lower expression in *PARK2*-mutant glial cultures compared with HD (Figure 7).

**Figure 8:**
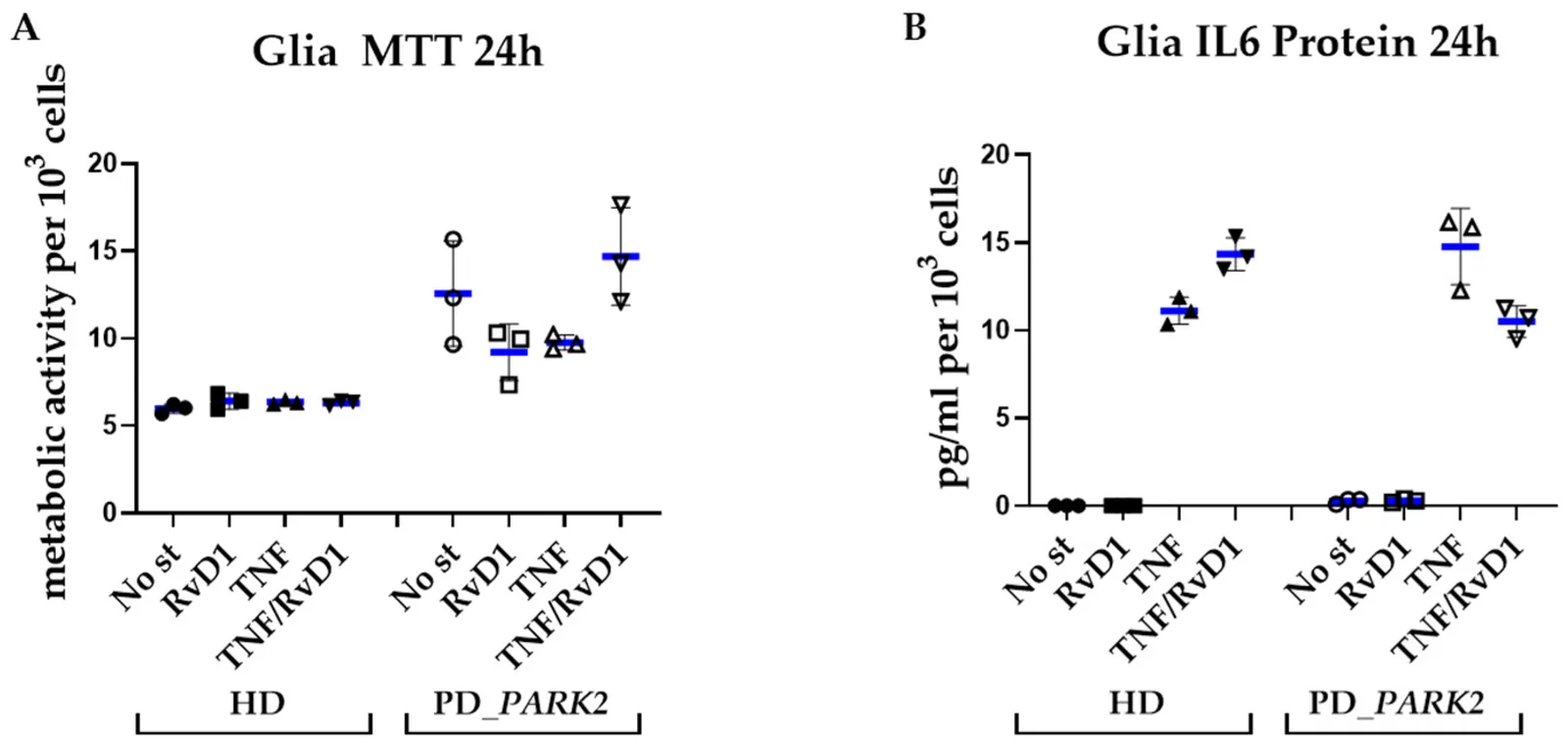
The effect of resolvin D1 on glial cell cultures: Pilot experiment included 2 cell lines—HD (IPSHD1.1S) and PD with mutation in the *PARK2* gene (IPSPDS14). Glial cells were cultured in the presence of RvD1 (20 ng/mL) for 24 h, either under basal conditions or following induction of inflammation by TNF-α (10 ng/mL). (**A**) MTT assay results. (**B**) IL-6 protein concentrations in glial supernatants, ELISA. Data are from three independent experiments performed with each cell line and are presented as mean ± SD, with the mean value for the group indicated in blue. Statistical analysis was not performed because of the limited number of cell lines in both groups.

Next, we conducted a pilot experiment to evaluate the effects of RvD1 using one HD and one *PARK2*-mutant glial cell line. Glial cells were cultivated in the presence of RvD1 (20 ng/mL) for 24 h, either under basal conditions or following induction of inflammation by TNF-α (10 ng/mL). RvD1 did not induce any apparent cytotoxic effects in glial cells. According to the MTT assay, increased MTT reduction was observed in stimulated *PARK2*-mutant glial cells cultured in the presence of RvD1, suggesting an RvD1-associated increase in cellular metabolic activity. Different responses to RvD1 were also observed for IL-6 protein secretion in TNF-α-stimulated cell cultures: IL-6 secretion increased in the HD cell line, whereas it decreased in the *PARK2*-mutant cell line (Figure 8). Given that only one cell line of each genotype was used, these observations cannot be generalized to HD and *PARK2*-mutant glia. However, these pilot data support further investigation of the effects of RvD1 on glial cells using additional independent cell lines.

## 4. Discussion

Nonresolving long-lasting inflammation is not a direct initiator of PD, but it contributes significantly to the disease pathogenesis [15,38,39]. Neuronal death induces an “eicosanoid storm,” including the production of prostaglandins and leukotrienes, which in turn initiates a robust inflammatory response. At a certain stage of the inflammatory response, the cascade of arachidonic acid (AA) and PUFA switches to the resolution phase, when specialized pro-resolving mediators (SPMs) are produced and stimulate macrophage-mediated clearance of debris, downregulate NF-κB, and attenuate pro-inflammatory cytokine production [9,39,40,41]. Resolution may fail if the secretion of resolution-driving factors or the expression of receptors for these molecules is reduced. Long-lasting neuroinflammation is characterized by deficiencies in resolution mechanisms, on the one hand, and by excessive activation of pro-inflammatory pathways, on the other hand [42]. Nonsteroidal anti-inflammatory drugs (NSAIDs) have therefore been investigated for their potential role in PD prevention and treatment. However, despite a recently published meta-analysis indicating a correlation between the use of several groups of NSAIDs and a reduced risk of developing PD [23], there is still no consensus regarding the effect of these drugs on neurodegeneration [23,43,44]. Multiple studies have shown that cyclooxygenase-2 (COX-2) is upregulated in DA neurons of the substantia nigra in PD patients and in experimental models and is a well-known therapeutic target for NSAIDs [45,46]. High activity of COX-2 leads to an excessive production of a potent pro-inflammatory prostaglandin E2 (PGE2) that might result in the death of DA neurons [43]. At the same time, PGE2 is necessary for the switch to SPM synthesis and timely resolution; for this reason, COX-2 inhibitors, despite their anti-inflammatory effect, disrupt endogenous resolution mechanisms [47,48]. In this regard, the issue of finding anti-inflammatory molecules that can neutralize the side effects of NSAIDs or act as independent anti-inflammatory agents in PD is highly relevant. SPMs may represent promising therapeutic agents, since they exert potent biological effects at low concentrations in experimental models of inflammation-associated diseases [40,49]. Nevertheless, it is not known which pro-resolving lipid mediator would be most effective in specific models of PD [39].

Data from the Human Protein Atlas (HPA) also provide information on the expression of the receptors for resolvin E1, BLT1 (*LTB4R*) and CHEMR23 (*CMKLR1*), and resolvin D1, GPR32 (*GPR32*) and FPR2/ALX (*FPR2*), in the human CNS. *LTB4R*, *CMKLR1*, and *FPR2* transcripts are detected in different human brain regions, with relatively low regional specificity. Brain single-nucleus data also show *CMKLR1* and *FPR2* expression in CNS macrophage populations, although their cell-type expression patterns differ [50]. Although *GPR32* is currently classified as not detected in the human brain in the HPA, a recent study demonstrated its expression in human cortical neurons and microglia, while it was nearly absent in astrocytes [30] and, in an earlier study, *GPR32* mRNA was also detected in primary human astrocyte cultures [51]. Overall, the available HPA data support the presence of several resolvin receptors in the human CNS and also indicate cell-type-specific differences in their expression. Multiple studies have confirmed the expression of SPM receptors in neurons and astrocytes, which determines the responsiveness of these cells to the anti-inflammatory effect of resolvins [13,52]. However, there are no data specifically on cells from PD patients with mutations in the *PARK2* gene. Meanwhile, it is well-established that the protein product of this gene, Parkin, significantly contributes to the development of neuroinflammation. The dysregulated process of mitochondrial clearance in Parkin deficiency causes the release of mitoDAMPs that trigger inflammation [53]. Due to its ubiquitin ligase activity, Parkin is directly related to NF-κB signaling and the function of SPM receptors as well. Wang et al. found that Parkin promotes NF-κB activation through K63 ubiquitination of RIPK1, resulting in increased p65 nuclear translocation [24]. Another substrate for ubiquitination by Parkin, GPR37, is the receptor for one of the SPM members, neuroprotectin D1 [34,54]. GPR37 deficiency was associated with increased IL-1β expression and impaired anti-inflammatory cytokine responses [34].

Considering the direct influence of Parkin dysfunction on inflammatory signaling, we carefully examined the expression of RvE1 and RvD1 receptors in neuronal and glial cell cultures of *PARK2*-associated PD patients compared to HDs, since specific studies addressing this issue are currently lacking. We also compared these data with the inflammatory status of each cell type, neurons and glia. Since both neurons and glial cells express enzymes involved in SPM biosynthesis, we also assessed the expression of the genes encoding 5- and 15-lipoxygenases, *ALOX5* and *ALOX15* [13]. Several published studies emphasize the dual role of these enzymes which, in conjunction with each other, generate SPMs [47,49,55].

Importantly, a dual nature is characteristic not only of inflammation in general but also of its particular mechanisms at different levels. Several studies classify 5-LOX as a key pro-inflammatory enzyme and 15-LOX as being responsible for the formation of pro-resolving mediators [56], whereas other work has demonstrated that 5-LOX deficiency leads to reduced SPM levels and impaired resolution of inflammation after myocardial infarction in mice [57]. Interestingly, 15-LOX is also capable of catalyzing all enzymatic steps without the need for 5-LOX [58]. It is known that the interaction of CHEMR23 with chemerin results in the increase in pro-inflammatory gene expression; the same effect is observed with the binding of leukotriene (LT) B4 to the BLT1 receptor. RvE1 interacts with both receptors in different ways. It acts as an agonist of CHEMR23, reducing TNF-α-induced NF-κB signaling, while at BLT1 it acts as a partial agonist and attenuates LTB4-induced signaling, including calcium mobilization and NF-κB activation [15,59,60,61]. Therefore, these two receptors modulate neuroinflammation depending on the ratio of pro-inflammatory and pro-resolving mediators in the intercellular environment. In contrast, RvD1 signaling via its receptors GPR32 and FPR2 mainly mediates anti-inflammatory and pro-resolving actions in neuroinflammation [15,62].

According to our results, in *PARK2*-mutant neurons, mRNA expression levels of resolvin receptors were generally comparable to those in HD neurons, with only *FPR2* expression showing a slight but statistically significant decrease. Interestingly, the pattern appeared to differ between *PARK2*-mutant and PD_OGM cells. In particular, PD_OGM neurons tended to exhibit higher expression of all four receptor genes. In *PARK2*-mutant glial cells, no significant changes in resolvin receptor gene expression were observed compared with HD cells, whereas PD_OGM glia showed a tendency toward higher *BLT1* and lower *CHEMR23* expression. Although these differences were not statistically significant, they may indicate that resolvin receptor expression is influenced by mutations in different genes. At the protein level, the most prominent finding was a significantly higher level of CHEMR23 in *PARK2*-mutant neurons compared with HD neurons. This was accompanied by decreased expression of genes encoding mPGES-2 (*PTGES2*), NF-κB component RELB, and the main pro-inflammatory cytokine *IL1B*, i.e., *PARK2*-mutant neurons demonstrated signs of less activated inflammatory profile compared to HD. These findings corresponded to a recent report on the anti-inflammatory function of RvE1/CHEMR23: in blood and adipose tissue of individuals with gain-of-function CHEMR23, IL-6 level was downregulated; whereas, mice lacking ChemR23 expression showed increased IL-6 levels [63].

To date, relatively little is known about the inflammatory intracellular signaling of neurons in neurodegeneration. Our recently published data also indicated the presence of a specific (hypo-activated) inflammatory status of *PARK2*-mutant neurons, when affected by glial soluble factors [19], which corresponded to the present results obtained on neurons in the absence of glial influence. Importantly, it was shown that in the initial phase of plaque formation in AD, Aβ-burdened neurons, but not glia, acted as inflammatory agents [64]. Activated glia, in turn, release pro-inflammatory factors and can induce the death of DA-neurons [65]. Moreover, *PARK2*-mutant glia exhibit pro-inflammatory shift compared to HD cells [27]. Therefore, these observations support targeting dysregulated inflammatory status of glial cells in *PARK2*-associated PD. Importantly, in neurodegenerative disorders, the coexistence and interplay of multiple pathogenic mechanisms, including distinct proteinopathies, disrupted mitochondrial homeostasis, and dysregulated neuroinflammation, underscore the need for complementary therapeutic strategies aimed at slowing disease progression [66], including approaches that promote the resolution of inflammation.

In our pilot experiment, RvD1 produced different responses in the two glial cell lines under TNF-α stimulation. In the *PARK2*-mutant cell line, RvD1 reduced IL-6 protein secretion and increased MTT reduction, the latter indicating increased cellular metabolic activity, whereas neither of these effects was observed in the HD cell line. Importantly, because the differentiation protocol used here yielded microglia-free glial cultures, the observed inflammatory changes reflect astroglia responses in the absence of a microglial contribution. However, since only one HD and one *PARK2*-mutant cell line were examined, these observations cannot be generalized to *PARK2*-associated PD. These pilot data support further investigation of the effects of RvD1 on glial cells using additional cell lines with different *PARK2* mutations.

## 5. Study Limitations

For this study, we used donor-matched neuronal and glial cultures derived through a common neural progenitor stage from iPSCs of healthy donors and PD patients carrying different mutations in the *PARK2* gene. Generation of patient-specific iPSC-derived neuronal and glial cell lines is technically challenging, and individual pathogenic *PARK2* variants are relatively rare, which limits the number of cell lines available for analysis. Therefore, the effects of individual *PARK2* variants were not analyzed separately, and we focused mainly on common features associated with *PARK2* mutations. This approach may be more relevant for the search for potential therapeutic strategies, since division into very small variant-specific groups would be less practical for this purpose. The number of available cell lines also limits the statistical power of between-group comparisons and the reliable assessment of data distribution. In addition to statistically significant differences, we also considered trends in gene expression, which should be interpreted with caution. Finally, an in vitro system cannot fully reproduce the complex cellular and molecular environment of the human brain. At the same time, this model allows us to examine cell-type-specific features associated with mutations in PD-related genes, which provides a basis for further studies using more complex experimental models. The glial cultures generated through neural progenitor-based differentiation are not expected to contain microglia, allowing the astroglia component of the neuroinflammatory response to be examined independently of microglial contribution, which represents an advantage of the model.

## 6. Conclusions

Parkinson’s disease involves multiple pathogenic mechanisms, which makes it important to explore different therapeutic strategies, including those that aim to restore resolution of inflammation. To our knowledge, this is the first study to characterize RvE1 (BLT1 and CHEMR23) and RvD1 (GPR32 and FPR2) receptor expression at both the mRNA and protein levels in iPSC-derived neuronal and glial cultures carrying *PARK2* mutations. The presence of these receptors indicates that they may represent targets for resolvin-based therapeutic approaches in *PARK2*-associated PD. We identified cell-type-specific receptor expression in *PARK2*-mutant cells, and also observed trends toward distinct expression patterns in PD cells carrying mutations in other genes, including GBA1 and LRRK2, which may suggest an influence of genetic background on resolvin receptor expression. *PARK2*-mutant neurons exhibited a less inflammatory profile compared with HD cells, in contrast to the pro-inflammatory shift we previously reported in *PARK2*-mutant glial cultures. These findings highlight the importance of taking into account both cell-type- and PD gene-associated differences in the development of resolvin-based therapeutic approaches for genetic forms of PD. Our pilot data further support the need for further studies of the potential anti-inflammatory effects of RvD1 in *PARK2*-mutant glial cells using additional *PARK2*-mutant glial cell lines.

## Figures and Tables

**Table 1:** Cell lines description. HD, healthy donors; PD, patients with Parkinson’s disease.

№	Disease Status	Donor	Genotype	iPSC Cell Line Symbol
1	HD	Healthy male, 60 years	normal	IPSRG2L
2	HD	Healthy female, 18 years	normal	IPSHD1.1S
3	HD	Healthy female, 26 years	normal	IPSFD3.9L
4	PD	PD male, the disease onset—40 years, biopsy 54 years	*het EX2 dup PARK2*	IPSPDS12
5	PD	PD female, the disease onset—30 years, biopsy 41 years	*hom EX8 del PARK2*	IPSPDS13
6	PD	PD male, the disease onset—38 years, biopsy 40 years	*het EX2 del PARK2*	IPSPDS14
7	PD	PD male, the disease onset—44 years, biopsy 60 years	*GBA* mutation (N370S)	IPSPDSG1.1
8	PD	PD male, the disease onset—50 years, biopsy 63 years	*LRRK2* mutation (G2019S)	IPSPDL2.15L

**Table 2:** The sequences of primers used in the study.

Gene	Forward	Reverse	Annealing t °C
*ALOX15*	TGTGAAAGACGACCCAGAGC	GGTCCCGAGCCTGTAAAGAG	63
*ALOX5*	CTGCTGTGCACCCCATTTTC	GCGTTGGCCTTGTCAAAGAG	63
*BLT1*	ATCATCCTGCTGTCAGTGGC	GAGCGGTCTAGACTCATGGC	55
*CHEMR23*	CTGTCCACACCTGGGTCTTC	GGTTGAGTGTGTGGTAGGGG	55
*IL1B*	GCTCGCCAGTGAAATGATGG	GTCCTGGAAGGAGCACTTCAT	60
*IL6*	CCTTCCAAAGATGGCTGAAA	CAGGGGTGGTTATTGCATCT	60
*FPR2*	TGGTAATGTGGCCGTGAAAGA	AGTCTGCTGGCTACACTGTTC	65
*HMBS*	GGCAATGCGGCTGCAA	GGGTACCCACGCGAATCAC	60
*GPR32*	AGCATGAAATCGGCAAGGGC	TCCATTGTCGTCGGAGTGCT	60
*NFKB1*	TTTTCGACTACGCGGTGACA	GTTACCCAAGCGGTCCAGAA	60
*NFKB2*	GCGTTGTCAACCTCACCAAC	CCGCCAGGTGTACTGGATAC	60
*PTGES2*	CAGAAGCCGAATCTCGCTGA	TCAGGTCATCGAACGCATCC	55
*RELA*	AGCGTGGGGACTACGACCTG	TTGCGAAAAGGAGCCTCGGG	60
*RELB*	GACCTCTCCTCACTCTCGCT	TGATGTACTCGTCGATGATCTCC	60
*18S rRNA*	CGGCTACCACATCCAAGGAA	GCTGGAATTACCGCGGCT	60

## Data Availability

The original contributions presented in this study are included in the article. Further inquiries can be directed to the corresponding authors.

## Abbreviations

PD	Parkinson’s disease
SPMs	specialized pro-resolving mediators
HD	healthy donor
Rv	resolvin
PUFAs	polyunsaturated fatty acids
DHA	docosahexaenoic acid
EPA	eicosapentaenoic acid
iPSCs	induced pluripotent stem cells
NSAIDs	nonsteroidal anti-inflammatory drugs
NPs	neural progenitors
